# Be Careful What You Eat!

**DOI:** 10.4269/ajtmh.19-0595

**Published:** 2019-09-23

**Authors:** Philip J. Rosenthal

**Affiliations:** Department of Medicine, University of California, San Francisco, California

The readership of the *American Journal of Tropical Medicine and Hygiene* is well acquainted with the risks of infectious diseases acquired from foods contaminated with pathogenic viruses, bacteria, protozoans, or helminths due to improper hygiene. Less familiar may be uncommon infections associated with ingestion of unusual uncooked foods, eaten either purposely or inadvertantly. A number of instructive examples have been published in the Journal within the last 2 years; these all involve helminths for which humans are generally not the definitive host, but can become ill when they unwittingly become accidental hosts after ingestion of undercooked animal products.

This issue of the Journal includes two reports on cases of sparganosis, which is caused by infection with larvae of *Spirometra* canine or feline tapeworms. One case involved a Chinese woman who presented with a brain mass^1^; another was a Thai resident of Switzerland who presented with a chronic history of subcutaneous nodules.^2^ In both cases, surgical exploration led to the unexpected discovery of living (mobile) worms, and genetic and morphological analyzes identified these as *Spirometra*. Both patients appeared to respond to surgical resection without antihelminthic therapy. Risk factors for infection were frequent ingestion of potentially undercooked stir-fried frogs for the Chinese patient and untreated tap water for the Thai patient.

*Angiostrongylus cantonensis*, the rat lungworm, is a roundworm of rodents that can be acquired after ingestion of larvae within slug, snail, or other invertebrate intermediate hosts. In humans, *A. cantonensis* can migrate to the brain, causing eosinophilic meningitis. *Angiostrongylus cantonensis* was originally described in Southeast Asia and the South Pacific, but it is now appreciated to cause disease in much of the tropics, including tropical areas of the Americas and Australia. Of relevance to American clinicians, the worm is endemic in Hawaii. A new report in the Journal describes 82 cases of *A. cantonensis* infection reported in Hawaii from 2007 to 2017; 51 of these cases were confirmed, the others probable.^3^ The most common presentations included headache, arthralgia, myalgia, and stiff neck in those older than 10 years, and fever and vomiting in children. Cerebrospinal fluid commonly demonstrated eosinophils. Of the 82 cases, 65 were hospitalized and two died. One case occurred after ingesting a slug on a dare, but most were probably due to ingestion of larvae associated with slugs or snails in unwashed produce. Travelers to Hawaii and other tropical areas, even in developed countries, should take care to wash produce before ingestion. And they should avoid ingesting uncooked slugs or snails.

*Angiostrongylus cantonensis* infection was also reported in the Journal recently in a Korean woman and her adult son, both of whom presented with eosinophilic meningitis after ingestion of live centipedes.^4^ Centipedes purchased from the same market used by the patients contained *A. cantonensis* larvae; thus, in addition to slugs, snails, and some other studied invertebrates, centipedes may be an intermediate host for the parasite. The patients appeared to respond to treatment with albendazole and dexamethasone. The value of treatment, which might exacerbate meningitis due to dying worms, has been considered uncertain; a recent perspective also published in the Journal suggested that treatment early after presentation with disease is advisable to prevent progression of illness, including migration of worms to the lungs.^5^ Ingestion of raw centipedes is best avoided.

A 15-year-old Thai girl presented with a 3-week history of migrating, erythematous edema of her right shoulder, ear, and face.^6^ Evaluation showed eosinophilia and a positive western blot for a 24-kDa antigen of the nematode *Gnathostoma spinigerum*, the cause of gnathostomiasis. Despite therapy with albendazole, the process recurred, with marked periorbital swelling. The problem resolved after treatment with ivermectin. The infection was presumably acquired by ingestion of undercooked freshwater fish, which the patient ate often.

A 45-year-old Nepali woman presented with dyspnea, chest pain, fever, and sweats of several weeks duration.^7^ Her recent medical history included a humerus fracture 2 months earlier, for which she ingested raw live slugs (“chiple kira”), a traditional remedy to accelerate bone healing. Investigation showed marked eosinophilia, an elevated erythrocyte sedimentation rate, and a right-sided pleural effusion on chest X-ray. The patient was treated with ivermectin and diethylcarbamazine for suspected parasitic infection. After a modest response to therapy, the patient worsened, with migratory skin lesions. A CT scan showed pericardial and bilateral pleural effusions and hypodense subcapsular tracks in the liver suggestive of visceral larva migrans. Paragonimiasis was diagnosed based on serology positive for *Paragonimus westermani* and identification of eggs in sputum. The patient responded well to treatment with praziquantel.

A 30-year-old American man presented with recurrent abdominal pain after ingesting home-cured salmon gravlax.^8^ The patient and family members described an esophageal wriggling sensation after gravlax ingestion. The patient underwent emergent exploratory laparotomy. An intussusception was identified in the mid-ileum, and a small bowel resection with primary anastomosis was performed. Surgical pathology showed eosinophilic inflammation and structures suggestive of *Anisakis* larvae. The patient’s pain recurred, and he was treated with albendazole. Two family members were then also treated with albendazole, and one rectally passed a 1.5-cm roundworm suggestive of *Anisakis*. Although sushi is widely enjoyed with low risk, anisakiasis remains a concern, especially after preparation by inexperienced sushi chefs.

A 58-year-old Korean woman presented with malaise and preprandial epigastric discomfort. Imaging showed a 7-cm hepatic mass.^9^ Biopsy of the mass demonstrated an eosinophilic abscess. Serology was negative for multiple helminths, but positive for *Toxocara canis*, a canine roundworm that can cause visceral larva migrans when eggs or larvae in animal tissue are ingested by humans. After treatment with albendazole, skin rash, increased eosinophilia, and increased size of the hepatic mass were noted, and the patient underwent hepatic lobectomy. The patient reported a history of yearly consumption of a cup of raw roe deer blood for perceived health benefits. Ingestion of raw animal blood is best avoided.

A 37-year-old man who resides in a rural area of Georgia, United States extracted a hair-like structure from a buccal lesion after a 7-month history of recurrent buccal “zig zagging blisters” and a 3-month history of nausea and vomiting.^10^ The structure was identified based on morphology as *Gongylonema pulchrum*, a nematode that has been reported to infect a number of animals, including humans. The patient was treated with a 3-day course of albendazole, but buccal worms persisted; symptoms resolved and did not recur after a 30-day course of albendazole. *Gongylonema pulchrum* can infect many animals, including cattle, dogs, and cats; intermediate hosts are cockroaches and dung beetles. After ingestion in humans, larvae burrow through the stomach or duodenal mucosa, followed by migration to the esophagus or oral cavity. Patients typically remove worms from their mouth mucosa after sensing movement. In this patient, *G. pulchrum* was likely acquired from inadvertent ingestion of cockroaches, which had infested grain that he stored in his double-wide trailer before consumption.

In another report in the Journal, six patients in Barcelona had infestations with *Dicrocoelium dendriticum*.^11^ *Dicrocoelium dendriticum*, the lancet liver fluke, is a parasite of cattle and other grazing animals. The organism can be acquired by humans by accidentally ingesting ants, an intermediate host. Infected humans can develop dicrocoeliasis, with biliary disease. More commonly, *D. dendriticum* eggs identified in human stool samples likely represent pseudo-infection, which is seen in individuals who ingest undercooked liver contaminated with *D. dendriticum*.

Unusual worm infections, as described recently in the pages of the Journal, are fascinating because of their curious biology and insights into human behavior. These infections can cause serious illnesses, and it is important to maintain suspicion for unusual pathogens in patients with relevant clinical presentations and risk factors. Some of these risk factors may only be elicited with a careful history for consumption of undercooked animal products.
